# Usability of Wearable Devices With a Novel Cardiac Force Index for Estimating the Dynamic Cardiac Function: Observational Study

**DOI:** 10.2196/15331

**Published:** 2020-07-21

**Authors:** Po-Jen Hsiao, Chih-Chien Chiu, Ke-Hsin Lin, Fu-Kang Hu, Pei-Jan Tsai, Chun-Ting Wu, Yuan-Kai Pang, Yu Lin, Ming-Hao Kuo, Kang-Hua Chen, Yi-Syuan Wu, Hao-Yi Wu, Ya-Ting Chang, Yu-Tien Chang, Chia-Shiang Cheng, Chih-Pin Chuu, Fu-Huang Lin, Chi-Wen Chang, Yuan-Kuei Li, Jenq-Shyong Chan, Chi-Ming Chu

**Affiliations:** 1 Division of Nephrology Department of Internal Medicine Taoyuan Armed Forces General Hospital Taoyuan Taiwan; 2 Division of Nephrology Department of Internal Medicine Tri-Service General Hospital, National Defense Medical Center Taipei Taiwan; 3 Department of Life Sciences National Central University Taoyuan Taiwan; 4 Big Data Research Center Fu-Jen Catholic University New Taipei Taiwan; 5 Division of Infectious Disease and Tropical Medicine Department of Internal Medicine Taoyuan Armed Forces General Hospital Taoyuan Taiwan; 6 Division of Biostatistics and Medical Informatics, Department of Epidemiology School of Public Health National Defense Medical Center Taipei Taiwan; 7 Graduate Institute of Medical Sciences National Defense Medical Center Taipei Taiwan; 8 Graduate Institute of Life Sciences National Defense Medical Center Taipei Taiwan; 9 Department of Nursing University of Kang Ning Tainan Taiwan; 10 School of Nursing College of Medicine Chang Gung University Taoyuan Taiwan; 11 Department of Nursing Tri-Service General Hospital Taipei Taiwan; 12 Institute of Cellular and System Medicine National Health Research Institutes Miaoli Taiwan; 13 Division of Pediatric Endocrinology & Genetics Department of Pediatrics Chang-Gung Memorial Hospital Taoyuan Taiwan; 14 Division of Colorectal Surgery Department of Surgery Taoyuan Armed Forces General Hospital Taoyuan Taiwan; 15 Department of Biomedical Sciences and Engineering National Central University Taoyuan Taiwan; 16 Department of Public Health Kaohsiung Medical University Kaohsiung Taiwan; 17 Department of Public Health School of Public Health China Medical University Taichung Taiwan

**Keywords:** cardiac force, running, acceleration, physical activity, heart rate

## Abstract

**Background:**

Long-distance running can be a form of stress to the heart. Technological improvements combined with the public’s gradual turn toward mobile health (mHealth), self-health, and exercise effectiveness have resulted in the widespread use of wearable exercise products. The monitoring of dynamic cardiac function changes during running and running performance should be further studied.

**Objective:**

We investigated the relationship between dynamic cardiac function changes and finish time for 3000-meter runs. Using a wearable device based on a novel cardiac force index (CFI), we explored potential correlations among 3000-meter runners with stronger and weaker cardiac functions during running.

**Methods:**

This study used the American product BioHarness 3.0 (Zephyr Technology Corporation), which can measure basic physiological parameters including heart rate, respiratory rate, temperature, maximum oxygen consumption, and activity. We investigated the correlations among new physiological parameters, including CFI = weight * activity / heart rate, cardiac force ratio (CFR) = CFI of running / CFI of walking, and finish times for 3000-meter runs.

**Results:**

The results showed that waist circumference, smoking, and CFI were the significant factors for qualifying in the 3000-meter run. The prediction model was as follows: ln (3000 meters running performance pass probability / fail results probability) = –2.702 – 0.096 × [waist circumference] – 1.827 × [smoke] + 0.020 × [ACi7]. If smoking and the ACi7 were controlled, contestants with a larger waist circumference tended to fail the qualification based on the formula above. If waist circumference and ACi7 were controlled, smokers tended to fail more often than nonsmokers. Finally, we investigated a new calculation method for monitoring cardiac status during exercise that uses the CFI of walking for the runner as a reference to obtain the ratio between the cardiac force of exercise and that of walking (CFR) to provide a standard for determining if the heart is capable of exercise. A relationship is documented between the CFR and the performance of 3000-meter runs in a healthy 22-year-old person. During the running period, data are obtained while participant slowly runs 3000 meters, and the relationship between the CFR and time is plotted. The runner’s CFR varies with changes in activity. Since the runner’s acceleration increases, the CFR quickly increases to an explosive peak, indicating the runner’s explosive power. At this period, the CFI revealed a 3-fold increase (CFR=3) in a strong heart. After a time lapse, the CFR is approximately 2.5 during an endurance period until finishing the 3000-meter run. Similar correlation is found in a runner with a weak heart, with the CFR at the beginning period being 4 and approximately 2.5 thereafter.

**Conclusions:**

In conclusion, the study results suggested that measuring the real-time CFR changes could be used in a prediction model for 3000-meter running performance.

## Introduction

### Background

As part of the body’s activity, exercise is planned, repeated, and structured to improve or maintain physical health. Exercising regularly and frequently helps to prevent serious illnesses such as heart disease, cardiovascular disease, type 2 diabetes mellitus, and obesity. In recent years, activities such as marathons, triathlons, and road running have become popular sports throughout the world [1-4]. The benefits of endurance exercise for health, such as improving lipid profiles, blood glucose control, blood pressure control, and increased insulin sensitivity, may partly explain the increased participation in marathon races [1-4]. Additionally, marathon runners show higher levels of hardy personality (ie, a group of characteristics related to personal perception of control, commitment, and challenges) than the general population [5]. With regard to gender differences in participation and running performance, Nikolaidis et al [6] noted that the men were faster and older than women, whereas female participation increased disproportionately to that of men, resulting in a decrease in the male-to-female ratio. Most women marathon runners range from 30 to 34 years and most men are 40 to 44 years. Furthermore, the number of master participants increased at a greater rate than that of their younger counterparts [7]. In addition to coordination of the skeletal and muscular systems, running primarily depends on the cardiopulmonary functions of pumping blood oxygen throughout the body. Nevertheless, highly intensive, strenuous physical exercise could have a negative impact on the cardiovascular system [8-10]. Marathon running has become a popular sport, and sudden cardiac arrest is rare but still occurs in approximately 1:100,000 of the general population, with the most common causes being hypertrophic cardiomyopathy or atherosclerotic coronary disease [11]. The incidence of sudden cardiac arrest resulting in death was significantly higher during marathons than during half-marathons and more common among men than in women. Even in young populations, sudden cardiac death may occur during running [11-13]. In addition, the mean age of the nonsurvivors was clearly younger than that of the survivors [11]. The common etiologies of sudden cardiac death in young populations include conduction system abnormalities, focal myocarditis, hypertrophic cardiomyopathy, and arrhythmogenic right ventricular cardiomyopathy, further causing myocardial injury [11-14]. Wearable wireless devices can monitor and offer information about human physiology and increase clinical diagnostic accuracy [15-19]. Combining wearable technology and medicine can further help people to improve their health and quality of life. This technology provides a real-time monitoring system during physical activity, especially exercise [20-24]. To date, these wearable devices can only monitor parameters such as heart rate, respiratory rate, electrocardiography, VO_2_ max test, running distance, and running speed [20-26]. They are still unable to determine some indicators precisely, such as the dynamic cardiac function changes in the wearer during running.

### Objective

The relationship between cardiac function changes during running and running performance should be further explored. Using a wearable device established in mobile health (mHealth), we explored the potential correlations among 3000-meter runners with stronger and weaker cardiac function during running. The aim of our study was to investigate this issue.

## Methods

### Study Population

We performed an observational study that was approved by the human trial committee and institutional review board of the Tri-Service General Hospital, Taiwan (TSGH-IRB-1-104-05-147). Military academy students participated in the study from February 2015 to January 2016 in Taiwan. The study’s selection conditions were to volunteer to participate in the study and to have the ability to complete a 3000-meter empty-handed run. Education and training personnel assisted participants in completing questionnaire contents and in measuring physiological parameters. Informed written consent was obtained from the participants for publication.

### Wearable Device and Cardiac Force Evaluation

This study used the American product called the BioHarness 3.0 (Zephyr Technology Corporation). It is nonpenetrating and contains a 3-axis gyroscope and an accelerometer to distinguish directions in the x, y, and z axes. It has an accelerometer and gyroscope for measuring the angular velocity and a GPS function to provide the speed, distance, and location. It can measure and estimate basic physiological parameters, including heart rate, respiratory rate, acceleration, maximum oxygen consumption, and temperature; thus, it can monitor the body’s activity state during running. Furthermore, the device has great reliability and validity and is better than regular heart rate belts that measure only the heart rate [25]. Excellent quality evidence from a recent systematic review study confirmed that the BioHarness 3.0 device could provide reliable and valid measurements of the heart rate across multiple contexts. In addition, it demonstrated good consistency during gold standard comparisons, supporting the validity criterion [25-28].

In exploring related factors pertaining to BioHarness 3.0 parameters and long-distance running, the credibility of the parameters measured by the BioHarness 3.0 have been considered by numerous studies, with one focusing on the measurement of its reliability and validity with 20 healthy male subjects, which consisted of 10 for reliability and 10 for validity [27]. For validity, the oxime part of the BioHarness 3.0 was compared with that of the Finnish T31 coded transmitter (Polar Electro) [27]. Lin et al [28] conducted a survey on 10 super-marathon runners, separating them into two groups and testing the correlations of the running distance with different acceleration levels (3, 6, 8, 9, 10, and 12 km/h). The survey addressed the correlation between the running speed and the 3-axis acceleration gauge with the x, y, and z axes. The results revealed a negative correlation between the y-axis and running at any speed (*r*=–.89 to –.92, *P*<.001). These results were similar to this study’s results; the activity (*r*=–.214 to –.317, *P*<.001) and peak acceleration (*r*=–.203 to –.226, *P*=.002) revealed a negative correlation [28].

We further used the BioHarness 3.0 to calculate the cardiac force index (CFI), a new method of detecting the heart’s condition instantly and comprehensively without penetration using the following calculation: CFI = weight × activity / heart rate; the CFI of running / CFI of walking ratio is the cardiac force ratio (CFR) [29,30]. Based on these methods, we then further explored the prediction model of running performance during 3000-meter runs.

### Data Collection

Before starting the 3000-meter empty-handed run, participants were required to squat, stand up, and then jump to check the heart rate belt’s 3-axis acceleration gauge function, which determines participant movement status. To ensure the questionnaire data’s consistency and completeness, data collection was performed after the participants completed the test and received their physiological measurement data.

**Figure 1:**
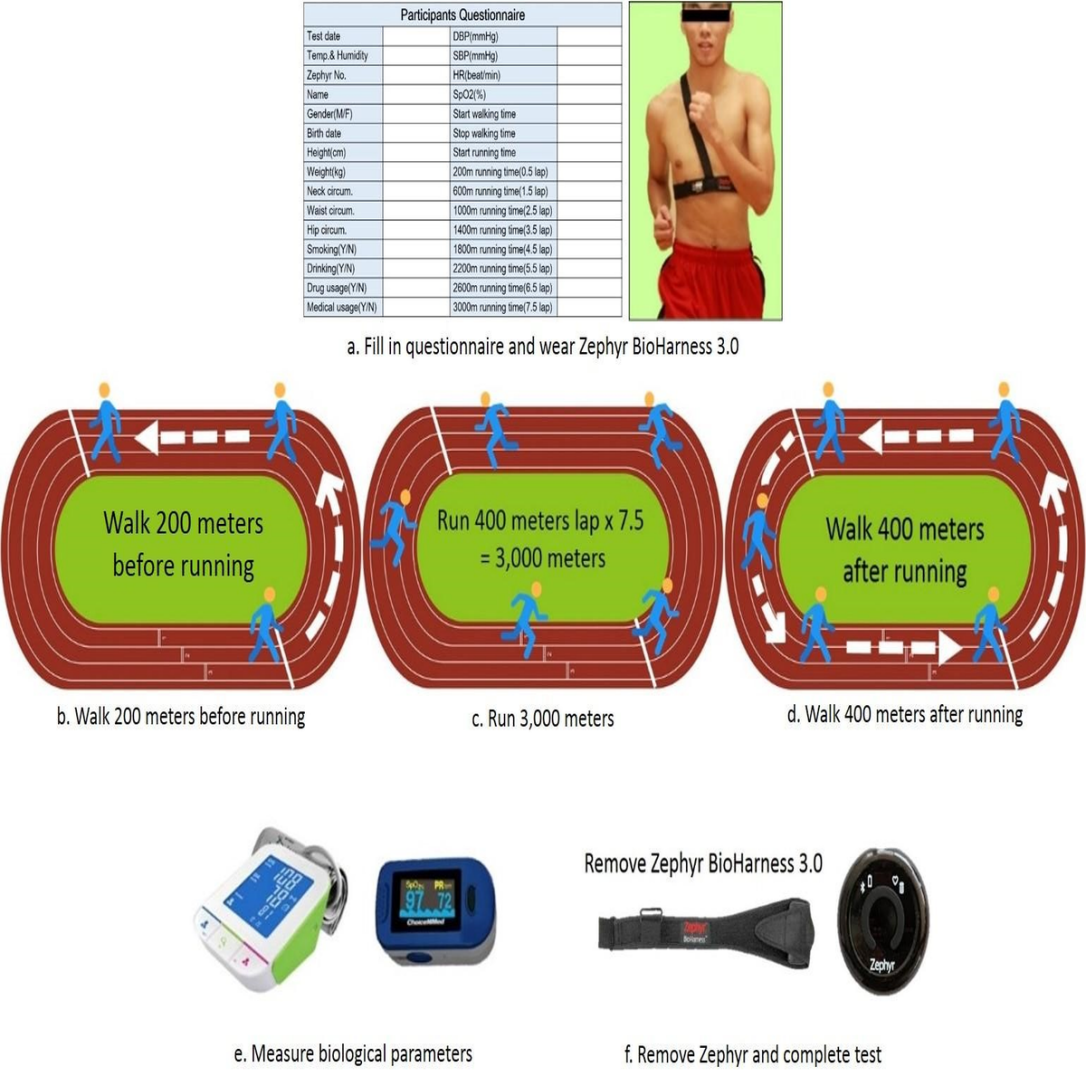
The process of collecting data followed six steps in this study.

Since completing a marathon race requires consistent strength training and an appropriate lifestyle, behaviors such as smoking, physical inactivity, and drinking are considered negative factors [31]. We also checked the baseline demography and associated lifestyle of all the runners in this study. The process of collecting data involved six steps (Figure 1):

- Participants were asked to complete the questionnaire with their name, gender, birth date, and personal health habits including smoking, drinking, drug usage, and medical history. Professional data collecting personnel completed the other blanks after testing the biological parameters and instructing participants in how to wear the BioHarness 3.0 heart rate belt correctly.
- Professional personnel led participants to the test starting position after confirming they were wearing the device correctly. Participants were asked to kneel, stand up, and walk at a normal speed in a half-circle (200 m) to the starting line.
- After participants got to the starting line, they were again asked to kneel and stand up. After ready and start commands from the leader, they began the 3000-meter running test (400 meter lap × 7.5 = 3000 meters) at the speed they see fit.
- After running the 3000 meters, participants were asked to jump once at the finish line and then walk another lap (400 m) at normal walking speed
- After walking the lap, participants were asked to rest until they are not gasping, at which time the professional personnel measured and recorded their biological parameters.
- After these measurements, the professional personnel removed the BioHarness 3.0, and the test was complete.

### Data Processing and Analysis

Because there are thousands of data points for individuals and manual artifact detection takes a great deal of time, we developed automatically identified data points (automatic filters) for when participants started to walk and run and when they ceased to walk and run. If the heart rate belt is worn improperly, automatic analysis can identify the equipment issues and delete the data. The Bioharness 3.0 can measure all sensor data precisely, such as the dynamic changes of heart rate with movement in the wearer during running (ie, dynamic cardiac function). The BioHarness 3.0 is snapped into an adjustable chest strap belt that contains skin conductive electrodes and captures the heart rate by recording the cardiac electric impulses and saving the output in beats per minute (bpm).

The BioHarness 3.0 parameters are defined as follows:

- Activity per second (ACi): measurement by the sensor of the amount of activity generated every second by the movement of human body parts. Activity level is measured by an accelerometer within the BioHarness 3.0
- Heart rate per second (HRi): number of heartbeats every second while exercise is measured by the sensor; the report from BioHarness 3.0 for the heart rate is in bpm and is provided as an average, at an interval of one second
- Cardiac force index activity (CFIi_AC): activity per second divided by heartbeats per second, where the (physical) activity and heart rate are estimated and measured by the BioHarness 3.0
- Cardiac force index peak acceleration (CFIi_PA): peak acceleration per second divided by heartbeats per second, where the peak acceleration and heart rate are estimated and measured by the BioHarness 3.0
- Cardiac force ratio activity (CFRi_AC): the CFI_AC value of running divided by the CFI_AC value of walking (at a speed of approximately 3 km per hour)
- Cardiac force ratio peak acceleration (CFRi_PA): the CFI_PA value of running divided by the CFI_PA value of walking (at a speed of approximately 3 km per hour)

After saving the analyzable data for variables such as heart rate, peak acceleration, activity per second, and so on in the BioHarness 3.0, the automation we developed calculates the CFIi_AC (the ith second CFI based on AC) and the CFRi_AC (the ith second CFR based on AC) and analyzes each variety of accumulated data according to the minute.

### Statistical Analysis

#### Data Collection

We described the gender, age, height, weight, BMI, neck circumference, waist circumference, hip circumference, hip-waist ratio, alcohol drinking habits, smoking habits, BioHarness 3.0 parameters, and CFI parameters by the number of subjects, percentage, mean, and standard deviation. Statistical analyses were conducted using SPSS Statistics version 20.0 (IBM Corp). All tests were 2-sided, and *P* values <.05 were considered statistically significant.

After the questionnaire collection was completed, the data were filed using Excel 2003 (Microsoft Corp) and coded according to the framework of this study. First, Excel Visual Basic for Application (Microsoft Corp) was used to distinguish among the collected data automatically in terms of the start of walking, end of walking, start of running, and end of running, which allowed for the identification of a cutoff point between walking and running. In addition, the questionnaire included a question on whether the heart rate belt was worn correctly. Therefore, the individuals whose belts were not worn appropriately were excluded after automatic analysis, while the analyzable data were saved. The CFIi_AC, CFIi_PA, CFRi_AC, and CFRi_PA were then calculated. Data analysis was conducted based on the research framework and purpose, which are described below.

#### Descriptive Statistics

The mean and standard deviation were used to describe continuous variables including age, height, body weight, BMI, neck circumference, waist circumference, hip circumference, waist-hip ratio, blood oxygen before and after the test, systolic pressure before and after the test, diastolic pressure before and after the test, heart rate before and after the test, activity per second, peak acceleration per second, heart beats per second, CFIi_AC, CFIi_PA, CFRi_AC, CFRi_PA, and the completion time of the 3000-meter empty-handed run.

The number and percentage are used to present the distribution of categorical variables including gender, medical history, medication history, smoking habits, drinking habits, and pass/fail results of the 3000-meter empty-handed run.

#### Inferential Statistics

Chi-square tests were used to determine the correlations between the 5 categorical variables (gender, medical history, medication history, smoking habits, and drinking habits) and the pass/fail results of the 3000-meter empty-handed run.

Independent sample *t* tests are suitable for comparing the means of two groups. The correlations between the 19 continuous variables (including the age, height, body weight, BMI, neck circumference, waist circumference, hip circumference, waist-hip ratio, blood oxygen before and after test, systolic pressure before and after the test, diastolic pressure before and after the test, heart rate before and after the test, activity per second, peak acceleration per second, heart beats per second, CFIi_AC, CFIi_PA, CFRi_AC, and CFRi_PA) and the pass/fail results of the 3000-meter empty-handed run were compared.

Multiple linear regression analysis was used to consider the effects of the two independent variables on dependent variables. For this method, the data type of the dependent variables must be continuous, while the data type of the independent variables can be continuous or categorical. Furthermore, through the establishment of a regression model, the prediction/forecast of dependent variables with independent variables could be achieved. Therefore, this study was intended to analyze 28 independent variables including gender, disease history, medication history, smoking habits, drinking habits, age, height, body weight, BMI, neck circumference, waist circumference, hip circumference, waist-hip ratio, blood oxygen before and after the test, systolic pressure before and after the test, diastolic pressure before and after the test, heart rate before and after the test, activity per second, peak acceleration per second, heart beats per second, CFIi_AC, CFIi_PA, CFRi_AC, and CFRi_PA. The dependent variable was the completion time for the 3000-meter empty-handed run.

In the case that there is only one independent variable, the analysis used is known as a simple logistic regression or univariate logistic regression. In a logistic regression analysis, the independent variables can be continuous or categorical. The purpose of using logistic regression is to establish a practical and reasonable model capable of providing the most concise and fit analysis results. Once established, the model can be used to predict the relationships between the dependent variable and a set of predictor variables. In this study, the logistic regression analysis was employed to establish a predictive model. The independent variables in the analysis included gender, medical history, medication history, smoking habits, drinking habits, age, height, body weight, BMI, neck circumference, waist circumference, hip circumference, waist-hip ratio, blood oxygen before and after the test, systolic pressure before and after the test, diastolic pressure before and after the test, heart rate before and after the test, activity per second, peak acceleration per second, heart beats per second, CFIi_AC, CFIi_PA, CFRi_AC, and CFRi_PA. The dependent variable was the pass/fail result of the 3000-meter empty-handed run.

Generalized estimating equations (GEEs) are primarily used to analyze the dependency of the data samples (including repeated measurement and long-term tracking studies). When the data are longitudinal and they record the state of a subject at different time points, the observation values from the same subject are all considered to have correlation. Since a subject might be tested more than twice in this study, the resulting data were interdependent. Therefore, the GEE was used in the study, and the covariance matrix was assumed to be AR(1). AR(1) indicated that the next time point was only highly correlated with the previous time point. The dependent variables were continuous variables (the results of the 3000-meter empty-handed run) and binary variables (pass or fail for the 3000-meter empty-handed run).

## Results

### Demographic Data

A total of 96 voluntary participants completed this study. The following sections show their basic demographic data, predictive physical factors for 3000-meter running performance, models of GEE and logistic regression for their running performance, and the stronger and weaker cardiac forces for the 3000-meter runners. Table 1 provides the descriptive demographic data: the average participant age was 23.25 [SD 3.48] years, average weight 69.40 [SD 9.34] kg, average BMI 22.93 [SD 2.66] kg/m^2^, average waist circumference 78.26 [SD 8.20] cm, average hip circumference 95.08 [SD 7.11] cm, and the average waist-to-hip ratio was 0.82 [SD 0.05]. The demographic characteristics were significantly correlated with the 3000-meter running time as follows: age (*r*=.241, *P*=.008), body weight (*r*=.233, *P*=.047), BMI (*r*=.284, *P*=.006), waist circumference (*r*=.319, *P*=.005), and waist-to-hip ratio (*r*=.30, *P*=.006). These variables correlated positively with the 3000-meter empty-handed running time.

**Table 1:** Descriptive demographic characteristics of participants (n=96).

Characteristic		Value, n (%)	Mean (SD)	Min-max
Gender, male		96 (100)	—	—
Age in years		96 (100)	23.25 (3.48)	19-38
Height (cm)		96 (100)	173.90 (6.42)	160-188
Weight (kg)		96 (100)	69.40 (9.34)	52-95
BMI (kg/m^2^)		96 (100)	22.93 (2.66)	18.18-31.02
Neck circumference (cm)		96 (100)	34.89 (2.79)	25-41
Waist circumference (cm)		96 (100)	78.26 (8.20)	62-102
Hip circumference (cm)		96 (100)	95.08 (7.11)	79-112
Waist-hip ratio		96 (100)	0.82 (0.05)	0.74-0.97
**Smoking**				
	Yes	12 (13)	—	—
	No	84 (88)	—	—
**Drinking**				
	Yes	7 (7)	—	—
	No	89 (93)	—	—
**Medication**				
	Yes	0 (0)	—	—
	No	96 (100)	—	—
**Medical history**				
	Yes	4 (4)	—	—
	No	92 (96)	—	—

### Prediction of Physical Factors Affecting Completion Times for 3000-Meter Run

To determine the factors that affected the 3000-meter completion times for the 96 valid cases, first we used BioHarness 3.0 parameters in the model, performing a univariate analysis with a simple linear regression to find the appropriate variate to build the 3000-meter running completion time model by performing a stepwise regression analysis on variates with a significant univariate (Table 2). According to the stepwise regression filtering, waist circumference, smoking, ACi1 (physical activity at the 1st minute), and CFRr.AC.w1i6 (CFI_AC.run at the 6th minute divided by CFI_AC.walk at the 1st minute) were significant factors in the completion times. Thus, the prediction model is as follows: 3000-meter running completion time = 1346.499 + 4.152 × [waist circumference] – 47.97 × [CFRr.AC.w1i6] – 5.43 × [ACi1] + 129.60 × [smoking]. From this formula, we know that with every increased centimeter on a participant’s waist circumference, the completion time increased by 4.152 seconds. The finishing times for smokers were 129.6 seconds more than for nonsmokers. For each unit increase in the CFRr.AC.w1i6, the running time decreased by 47.97 seconds, and for each unit increase in the ACi1, running time decreased by 5.43 seconds.

**Table 2:** Physical predictors of 3000-meter running qualification (univariate).

Variable	𝛽	SE	*P* value	95% CI
Constant	1346.50	207.146	<.001	935.03 to 1757.97
Waist circumference	4.15	1.260	.001	1.64 to 6.66
Smoking	129.60	30.950	<.001	68.13 to 191.07
CFRr.AC.w1i6^a^	–47.97	11.060	<.001	–69.94 to –25.99
ACi=1^b^	–5.43	1.370	<.001	–8.15 to –2.71

^a^CFRr.AC.w1i6: CFI_AC.run at 6th minute divided by CFI_AC.walk at 1st minute.

^b^ACi=1: activity per second accumulated until the 1st minute after the contestant started to run.

We used the BioHarness 3.0 parameters in the model and performed a univariate analysis with binary logistic regression. We then used a forward logistic regression method on the significant variates from the previous univariates to find the predictors affecting the 3000-meter qualification. We established a prediction model by multivariate logistic regression (Table 3). The results showed that the waist circumference, smoking, and ACi7 were significant factors for the 3000-meter run qualification. The prediction model was as follows: ln (3000 meters running performance pass probability / fail results probability) = – 2.702 – 0.096 × [waist circumference] - 1.827 × [smoke] + 0.020 × [ACi7]. If the smoking and ACi7 were controlled, contestants with larger waist circumferences tended to fail the qualification based on the formula above. If the waist circumference and ACi7 were controlled, smokers tended to fail more often than nonsmokers.

**Table 3:** Physical predictors of 3000-meter running qualification (multivariate logistic regression).

Variate	𝛽	SE	*P* value	OR^a^	95% CI
Waist circumference	–0.096	0.032	.003	0.909	0.853-0.968
Smoking	–1.827	0.890	.04	0.161	0.028-0.920
ACi=7^b^	0.020	0.007	.004	1.020	1.006-1.034
Constant	–2.702	4.185	.52	0.067	—

^a^OR: odds ratio.

^b^ACi=7: activity per second accumulated until the 7th minute after the contestant started to run.

### Comparison of Prediction Model and Actual Test Time of Finishing the 3000-Meter Run

The results showed no significant difference between the estimated time and actual completion time (*P*=.42), indicating no significant difference between the estimated completion time and actual measurement of the number of seconds in this study prediction model (Table 4).

**Table 4:** Comparison of linear model estimation results and actual measurements of finish times for 3000-meter runs (n=96).

Variate	Mean (SD)	SE	95% CI	*P* value
Linear model estimation	885.42 (79.16)	8.08	869.58-901.26	.42
Actual measurement	885.33 (124.61)	12.72	870.65-900.01	—

### Comparison of Pass/Fail Results Predicted by the Logistic Model With Actual Results of 3000-Meter Empty-Handed Run

A McNemar test was employed to compare the pass/fail results estimated using the logistic model with the actual results of the 3000-meter empty-handed run (Table 5). There was a statistically significant correlation between the estimated and actual passing probability (Pearson coefficient *r*=.477, Spearman correlation coefficient=.477, 𝜅=0.476 [SE 0.090, 95% CI 0.300-0.653], and the area under the receiver operating characteristic curve 0.768, 95% CI 0.687-0.857).

**Table 5:** Comparison of the pass/fail results of the 3000-meter empty-handed run predicted by the logistic model with actual measured results.

Logical model prediction	Actual measurement		McNemar	Correlation coefficient	
	Fail	Pass	*P* value	Spearman	*P* value
Fail	39 (40.63)	13 (13.54)	.84	.477	<.001
Pass	12 (12.50)	32 (33.33)	—	—	—

### Comparison of Stronger and Weaker Cardiac Forces During 3000-Meter Run

**Figure 2:**
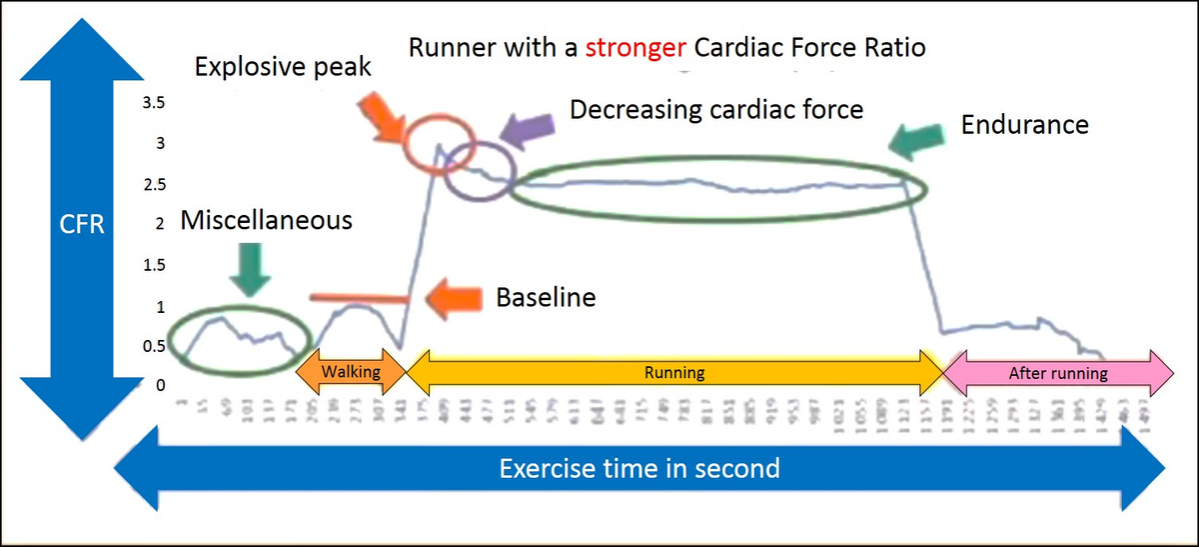
Runner with stronger cardiac force during 3000-meter running.

The relationship between the CFR of a healthy 22-year-old person with strong cardiac force and time is illustrated in Figure 2. In this example, a representative runner has a strong heart. The runner uses the method and apparatus for monitoring their cardiac status during exercise according to the protocol, including the walking period, running period, and postrunning period. During the running period, data are obtained while the user slowly runs 3000 meters. The postrunning period comes after the runner has stopped running. Figure 2 uses the maximum CFI of the walking period to serve as a reference to calculate the CFRs of other time periods. The relationship between the CFR and time is plotted. Thus, this finding shows the runner’s CFR varying with changes in activity. The signals before the walking period are noise. When the runner changes from the walking period to the running period, the runner converts from a relatively static walking condition to a dynamic running condition. Since the runner’s acceleration increases, the CFR quickly increases to an explosive peak, indicating the runner’s explosive power. This period shows a CFR of approximately 3. After the explosive peak, the CFR begins to decrease, and after a time lapse, it becomes moderate and gentle, entering an endurance period at approximately 2.5 CFR. After the running period has ended, the postrunning period begins. At this moment, the runner’s dynamic condition ends, acceleration decreases very quickly, and the CFR also decreases.

**Figure 3:**
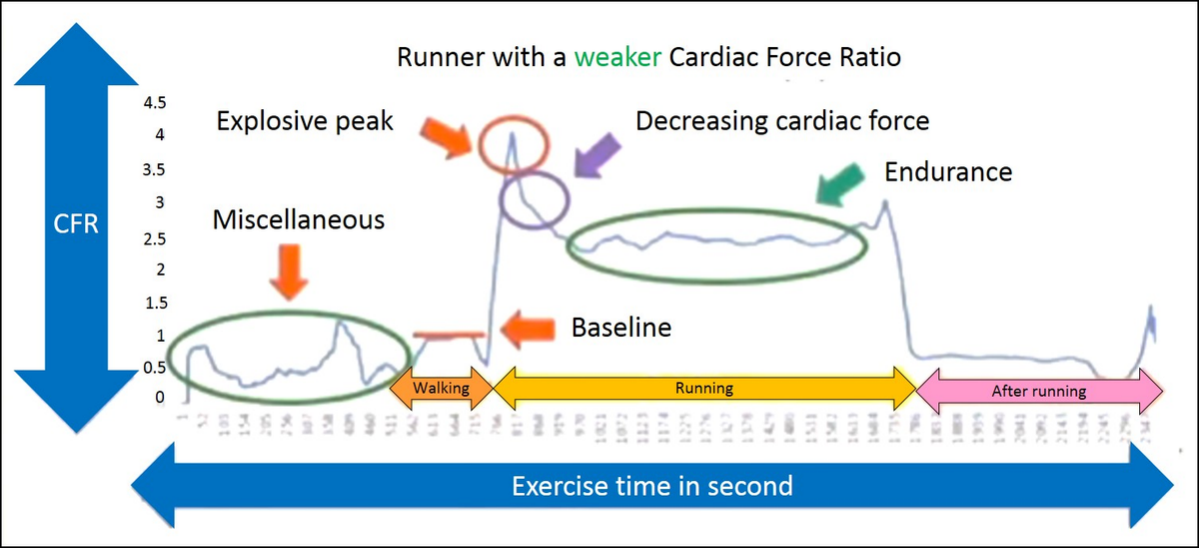
Runner with weaker cardiac force during 3000-meter running.

Another relationship, this time between the CFR of a 44-year-old with weak cardiac force and time is illustrated in Figure 3. The protocol for this runner is the same as that for the first runner (Figure 2), with the walking period, running period, and postrunning period. During the running period, data are obtained while the user slowly runs 3000 meters. The postrunning period occurs after the runner has stopped running. Figure 3 uses the maximum CFI of the walking period as a reference to calculate the CFRs of the other periods. The relationship between the CFR and the time is plotted. Signals from before the walking period are noise. Again, the runner’s condition changes from relatively static when walking to dynamic when running, and the CFR reaches an explosive peak with acceleration, at approximately 4. After the explosive peak, the CFR decreases and eventually becomes moderate and gentle, entering an endurance period at approximately 2.5 CFR. After the running period ends, postrunning period begins, the runner’s dynamic condition ends, acceleration decreases very quickly, and the CFR also decreases.

In summary, this study investigates a new calculation method for monitoring cardiac status during exercise that uses the CFI of walking for the runner as a reference to obtain the ratio of between the cardiac force of exercise and that of walking (CFR) to provide a standard for determining if the heart is capable of the exercise. Since the CFR is simply a ratio, it helps eliminate individual differences among the users and errors caused by changing the location of the detection units.

## Discussion

### Principal Findings

Previous traditional study methods used only parameters such as questionnaires and biochemical values to predict a runner’s completion time [27,28]. Our study results revealed that physical characteristics including age, body weight, BMI, waist circumference, and waist-to-hip ratio have impacts on the 3000-meter running qualification. However, this study uses a new method for detecting cardiac status that calculates the instantaneous CFI of the runner by using weight, heart rate, and acceleration or activity level of the user to transform the physiological parameters, which are not quite meaningful individually, into a CFI that is more meaningful. The CFI is provided for detecting the cardiac status of the runner in a dynamic manner. We successfully established a novel prediction model for the running performance based on the real-time cardiac force during running.

In this study, the data obtained from wearable technology were included as variables to explore the factors in the demographic characteristics, physiological parameters, and BioHarness 3.0 parameters that were correlated with the 3000-meter empty-handed run and its pass/fail results to establish a predictive model.

The summary of the results and discussion revealed that certain demographic characteristics in our study were significantly positively correlated with the completion time of the 3000-meter empty-handed run, including age, body weight, BMI, waist circumference, and waist-hip ratio. By contrast, there were no statistically significant correlations between physiological parameters and completion time of the 3000-meter empty-handed run. The BioHarness 3.0 parameters that were significantly negatively correlated with completion time of the 3000-meter empty-handed run included an ACi=1-10, PAi=1, PAi=8, PAi=9, PAi=10, HRi=1-10, CFRr.AC.w1i5, CFRr.AC.w1i6, CFRr.AC.w1i7, CFRr.AC.w1i8, CFRr.AC.w1i9, and CFRr.AC.w1i10.

Among the demographic characteristics examined in our study, the statistically significant variables for the pass/fail results of the 3000-meter empty-handed run included age, waist circumference, hip circumference, waist-hip ratio, and smoking. None of the physiological parameters were statistically significant variables for the pass/fail results of the 3000-meter empty-handed run. Among the BioHarness 3.0 parameters, statistically significant variables for the pass/fail results of the 3000-meter empty-handed run included an ACi=1-10, PAi=1, PAi=6, PAi=7, PAi=8, PAi=9, PAi=10, CFI.AC.WALK=4, and CFI.AC.RUNi=5-10. The HRi was not a statistically significant variable.

In the multivariate linear regression model, waist circumference, ACi=1, and CFRr.AC.w1i6 were able to predict the completion time of the 3000-meter empty-handed run. Moreover, there were no significant differences between the predicted values and the actual measured values. In the logistic regression model, waist circumference, smoking habits, and ACi=7 were able to predict the pass/fail results for the 3000-meter empty-handed run. The correlation between the estimated and actual passing probability was statistically significant.

Finally, a GEE-linear (binary) analysis was conducted on the individuals tested one or more times. The results show that the CFI.PA.WALK=3 was capable of predicting the completion time of the 3000-meter empty-handed run, while the ACi=3 and CFRr.AC.w1i7 were capable of predicting the pass/fail results of the 3000-meter empty-handed run.

### Exploration of Correlation Factors Between Demographic Characteristics and 3000-Meter Empty-Handed Run

#### Age

In 1988, Marti et al [32] explored the correlations between age, body weight, BMI, and lifestyle and 16-km running performance in 4000 joggers. The results of the study showed that age was a predictive factor for completion time of the 16-km run (β=.37, *P*<.001) [32]. In 2009, Leyk et al [31] analyzed 439,278 running times from a results lists of 108 marathon competitions. Their primary findings were there are virtually no relevant running time differences (*P*<.01) in marathon finishers from 20 to 55 years and the majority of middle-aged and elderly athletes have training histories of less than 7 years of running. Lara et al [33] explored the correlations of gender and age with completion time in marathon runners. The results showed that there was a significant positive correlation between the men’s age and completion time (Pearson correlation coefficient *r*=.92, *P*<.05). Knechtle et al [34] analyzed the correlation between age and marathon completion time in marathon runners aged 5 to 93 years. The results showed that completion time was increased with age in marathon runners over an age range of 5 to 93 years (Pearson correlation coefficient *r*=.97, *R*^2^=.94, *P*<.001) as well as marathon runners with an age range of 18 to 80 years (Pearson correlation coefficient *r*=.99, *R*^2^=.98, *P*<.001). The results of our study showed that age was significantly positively correlated with completion time of a 3000-meter empty-handed run (seconds) in males aged 19 to 38 years (mean age 23.25 [SD 3.48] years; Pearson correlation coefficient *r*=.241, *P*<.001). The result indicated that the older the males were, the longer it took them to complete the 3000-meter run.

#### Body Weight and Body Mass Index

The results of our study showed that body weight (69.4 [SD 9.34] kg) was significantly positively correlated with the 3000-meter empty-handed running performance (seconds; Pearson correlation coefficient *r*=.233, *P*<.001). This result indicated that the heavier the body was, the longer it took to complete the 3000-meter run. Previously, Marti et al [32] showed that the BMI value was a predictive factor for completion time (β=0.23, *P*<.001). The results of our study showed that BMI (22.93 [SD 2.66] kg/m^2^) was positively correlated with completion time of the 3000-meter empty-handed run (Pearson correlation coefficient *r*=.284, *P*<.001). This finding indicated that the larger the BMI was, the longer the completion time. Our result was consistent with the above literature.

#### Waist Circumference, Neck Circumference, and Hip Circumference

Recently, many studies have shown that neck and waist circumference are highly correlated and are related to the metabolic status of the body [35-37]. In our study, waist circumference was positively correlated with completion time of the 3,000-meter empty-handed run (Pearson correlation coefficient *r*=.319, *P*<.001). Namely, the larger the waist circumference was, the longer the completion time. Neck circumference was significantly negatively correlated with running performance in the GEE-binary analysis (OR 0.88; *P*=.03): the larger the neck circumference was, the more likely the individual would obtain a failing result. This finding was similar to the waist circumference result. In our study, hip circumference of the individuals who failed the 3000-meter empty-handed run test was larger than that of those who passed the run test (96.44 [SD 7.66] vs 93.55 [SD 6.17], *P*=.046), which was similar to the above results. The waist-hip ratio was also positively correlated with completion time (seconds) of the 3000-meter empty-handed run (Pearson correlation coefficient *r*=.300, *P*<.001), indicating that the larger the waist-hip ratio was, the longer the completion time.

### Exploration of Correlation Factors Between Bioharness 3.0 Parameters and 3000-Meter Empty-Handed Run

A study conducted by Johnstone et al [27] showed that the parameters collected by the BioHarness 3.0 have high reliability and validity. The reliability and validity were measured in 20 healthy males (10 subjects for a reliability analysis and 10 for a validity analysis). In terms of the validity of the BioHarness 3.0, blood oxygen measurement was assessed using the T31 (Polar Electro) as the standard while the respiratory rate measurement was assessed using a US-made face mask (Hans Rudolf Inc) as the standard. In addition, subjects performed incremental exercises (walking 4 to 6 kilometers per hour, jogging 8 to 10.5 kilometers per hour, and running 11 kilometers per hour) carrying the portable METAMAX 3B (Cortex Medical; weight 650 g). The heart rate, respiratory rate, and accelerometer were measured. The results showed that heart rate was statistically significantly correlated with the standard value (*r*=.98). Excluding the walking period, a strong correlation was observed between heart rate and the standard at a running speed of 8 to 10.5 kilometers per hour (*r*=.93). As running speed increased, the correlation between heart rate and the standard decreased (11 km/h, *r*=.67) [27]. The results of our study showed that the correlation between the accumulated heart rate per minute and completion time was reduced as running time was prolonged and running speed increased (HRi=1, *r*=–.283; HRi=10, *r*=–.258). Our results were similar to the results of the study described above. The correlation between the amount of activity measured using the BioHarness 3.0 and the standard was statistically significant (*P*<.001, *r*=.91).

In 2014, Lin et al [28] divided 10 ultramarathon runners with distinct performances into two groups and used accelerometers to measure the correlation between acceleration and running distance at different speeds (3, 6, 8, 9, 10, and 12 kilometers per hour). The study examined the correlations between the x, y, and z axes of the triaxial acceleration gauge and running speed. The results showed that the y-axis was negatively correlated with running speed at any speed (*r*=–.89 to –.92, *P*<.001). These results were similar to our results in that activity (*r*=–.214 to –.317, *P*<.001) and peak acceleration (*r*=–.203 to –.226, *P*=.003) were negatively correlated with completion time.

### Limitations

There are some limitations to this study. The subjects of this study were students (college-level and graduate-level) and service personnel at a military academy in the northern region (including voluntary and compulsory service personnel). The subjects were all males, and females were not included in the study. Since the wearable device must be worn tightly against the skin at the lower edge of the chest, most women were unwilling to participate. In addition, the poor availability of the output from the women’s physiological/structural relationship data rendered it impossible to include these data for analysis. This study only considered the subjects’ smoking, drinking, medication, and medical histories. Potential interference factors (exercise frequency, lifestyle, dietary habit, and environmental factors) were not explored in this study.

### Future Works

Our study only included one military college in the northern region as the research subject. We suggest that the sample size in future study be increased to include field troops and national army physical fitness evaluation centers. In addition, wearable technology can be used as one form of physical strength evaluation equipment for the national army to reduce the risk of sport injury and accidents. Completion time was correlated with exercise frequency, lifestyle, dietary habit, environmental factors, and caffeine intake. We suggest that future studies include the above factors as variables. Taking into the consideration the inconvenience among women in wearing the heart rate belt, we also suggest that the belt be worn on various parts of the body and reliability and validity be confirmed. In addition, the staff should confirm that the subjects wear the heart rate band tightly against the skin when collecting the data, which would improve the usability of the data.

In this study, smoking was included as a variable and discussed. The output parameters of the BioHarness 3.0 also include the respiratory rate. Since running is closely related to cardiopulmonary function, we suggest that future studies include the respiratory rate for in-depth exploration. In addition, the output parameters of the BioHarness 3.0 include posture, activity intensities on three different planes (sagittal plane, vertical, Z; cross-sectional plan, mediolateral, X; and longitudinal plane, anteroposterior, Y). We suggest that these parameters be included in subsequent studies for analysis.

We also suggest combining the relevant physiological values embedded in the heart rate belt and the CFI and CFR values discussed in this study with a mobile app, which would allow subjects to understand their own exercise status in real time and provide real-time data. We propose uploading the CFI and CFR obtained in this study to the cloud community for people to compare with themselves and others so the CFI and CFR may serve as incentive parameters for physical activity.

### Conclusions

This study used wearable technology and focused on real-time cardiac function changes related to 3000-meter running and qualification results. Based on the cardiac force, we successfully established a reliable prediction model for running performance. In the future, this cardiac force model can be used during running training and may assist in research on the application of the CFI.

## Abbreviations

ACi: activity per second
bpm: beats per minute
CFI: cardiac force index
CFIi_AC: cardiac force index activity
CFIi_PA: cardiac force index peak acceleration
CFR: cardiac force ratio
CFRi_AC: cardiac force ratio activity
CFRi_PA: cardiac force ratio peak acceleration
GEE: generalized estimating equation
HRi: heart rate per second
mHealth: mobile health
